# Consequences of post-discharge hospitalisation on the growth of young Bangladeshi children hospitalised with diarrhoea: a secondary case-control analysis of Antibiotics for Children with Diarrhea (ABCD) trial

**DOI:** 10.7189/jogh.15.04039

**Published:** 2025-02-14

**Authors:** Md Farhad Kabir, Irin Parvin, Abu Sadat Mohammad Sayeem Bin Shahid, Rina Das, Mst Mahmuda Ackhter, Tahmina Alam, Sharmin Khanam, Jannat Sultana, Shajeda Nasrin, Rumana Sharmin, Mohammad Tashfiq Ahmed, Mehnaz Kamal, Md Tanveer Faruk, Sharika Nuzhat, Farzana Afroze, Tahmeed Ahmed, Mohammod Jobayer Chisti

**Affiliations:** 1 International Centre for Diarrhoeal Disease Research, Bangladesh (icddr,b), Dhaka Bangladesh; 2 Bangabandhu Sheikh Mujib Medical University, Shahbag, Dhaka, Bangladesh; 3 Gangarosa Department of Environmental Health, Rollins School of Public Health, Emory University, Atlanta, Georgia, USA

## Abstract

**Background:**

Due to the scarcity of published data on growth among children with severe diarrhoea requiring readmission during post-discharge follow-up, we aimed to investigate the potential impact of post-discharge readmission at day-90 follow-up on growth in diarrheal children aged 2–23 months.

**Methods:**

We performed a secondary analysis using Bangladesh site data from the Antibiotic for Children with Diarrhea (ABCD) trial, a multi-country, randomised, double-blind, placebo-controlled study conducted from July 2017 to July 2019. Children aged 2–23 months who had severe diarrhoea defined as having acute diarrhoea with some/severe dehydration, or severe stunting, or moderate wasting, were admitted to the facility. In this analysis, we classified children who were re-hospitalised within a 90-day post-discharge follow-up period as cases and randomly selected controls who did not require re-hospitalisation, matching them by similar ages and sexes in a 1:3 ratio. We gathered anthropometric data on enrolment and day 90 follow-up. The outcome variables were changes in nutritional indicators height-for-age (ΔHAZ), weight-for-age (ΔWAZ), weight-for-height (ΔWHZ), and mid-upper arm circumference (ΔMUAC). We assessed for growth changes at day 90 post-discharge follow-up using multivariate linear regression.

**Results:**

Among 1431 diarrhoeal children enrolled, we identified 145 cases and 435 controls. In terms of the baseline admission characteristics, the cases were less likely to be immunised (81% vs. 72%; *P* = 0.031), vomit (11% vs. 22%; *P* = 0.001), and have dehydrating diarrhoea (26% vs. 36%; *P* = 0.026) than the controls. After adjusting for potential covariates, the cases had a significant reduction in growth than the controls at 90 days of post-discharge follow-up, according to anthropometric indices: ΔHAZ (*β* = −0.11; 95% confidence interval (CI) = −0.21, −0.01; *P* = 0.029), ΔWAZ (*β* = −0.24; 95% CI = −0.35, −0.14; *P* < 0.001), ΔWHZ (*β* = −0.25; 95% CI = −0.39, −0.12; *P* < 0.001), and ΔMUAC (for children 6–23 months, *β* = −0.17; 95% CI = −0.29, −0.04; *P* = 0.011).

**Conclusions:**

Diarrhoeal children aged 2–23 months requiring readmission during the 90-day post-discharge follow-up period had a significant deterioration of ponderal and linear growth, compared with those who did not require readmission. This finding underscores the importance of early identification of children with risks of post-discharge readmission and designing a package of post-discharge trials, including social and nutritional interventions that may help to reduce post-discharge readmissions as well as subsequent growth faltering.

Diarrhoea continues to be a significant worldwide public health challenge and one of the leading causes of death, particularly among children under the age of five, causing 1.7 billion cases and nearly half a million child deaths each year [1]. The burden is particularly severe in low- and middle-income countries (LMICs), especially in sub-Saharan Africa and Southeast Asia [2]. The child mortality rate in Bangladesh due to diarrhoea has reduced significantly over the years, but nevertheless remains a substantial contributor with a mortality rate of 15.88 deaths per 100 000 children in 2021 [3].

Potential linear growth faltering due to diarrhoea in early childhood is a critical concern in this sense [2,4–11]. The potential ramifications of paediatric diarrhoea, spanning both immediate and prolonged impacts on development, pose a substantial threat to the future well-being of children [8,11,12]. The condition impacts around 250 million children in LMICs each year, putting them at risk of impaired physical growth and development in early life [4,7,13–16]. Several studies have emphasised the connection between diarrheal diseases in early life and subsequent malnutrition [3–7], acknowledging the first two years of life as a critical window for growth and development [17,18]. For children under the age of five in Bangladesh, where malnutrition is prevalent, there is clear evidence of an association between diarrhoeal diseases and compromised growth, along with adverse cognitive developmental outcomes [7].

Children experience very rapid physical and cognitive development during the early years of life. This period is very sensitive for them, as many negative effects like illness, malnutrition, and other environmental stressors make them highly vulnerable and sometimes lead to fatal consequences [14]. Hospital admissions, especially with diarrhoea, dehydration, and/or malnutrition can have a serious impact on the health and well-being of children with potential and long-term consequences for health risks [13,19,20]. Children who require repeated hospitalisation due to various illnesses are at an even greater risk [17]. The possible impact of re-hospitalisation on the growth and development of children cannot be underestimated because it can lead to unwanted long-term consequences that may negatively affect their future [21].

The relationship of diarrhoea on children's health and well-being, including growth patterns, has been examined in prior research [7,8,12,14], but little emphasis has been placed on the impact of re-hospitalisation on linear and ponderal growth. This research gap emphasises the necessity to investigate the precise consequences of post-discharge hospitalisation due to diarrhoea or other illnesses on the linear and ponderal growth of children.

To better understand the long-term impacts of severe acute illness that led to post-discharge hospitalisation, it is imperative to understand the disparities in anthropometric outcomes of re-hospitalised children. We thus aimed to assess the potential consequences of post-discharge re-hospitalisations at a day-90 follow-up on the linear and ponderal growth in children aged 2–23 months.

## METHODS

### Study site

One segment of the multi-country Antibiotic for Children with Diarrhea (ABCD) trial was conducted in Bangladesh, at the International Centre for Diarrhoeal Disease Research, Bangladesh (icddr,b), Dhaka, Bangladesh, the world’s largest diarrheal treatment centre [22]. The hospital is situated in Dhaka, the capital city of Bangladesh, and provides free health care services to approximately 200 000 patients every year. The hospital predominantly provides treatment to all patients who are experiencing diarrhoea, with or without associated complications. The data for the trial was collected from its two treatment centres, Dhaka Hospital and Mirpur Treatment Centre.

### Study design and study participants

The ABCD trial was a multi-country, randomised, double-blind, placebo-controlled clinical trial conducted across 36 outpatient hospital departments or community health centres in seven countries, including Bangladesh, from 1 July 2017 to 10 July 2019 [23]. Young children aged 2–23 months who sought treatments in the health facilities for non-bloody diarrhoea were enrolled for this study. Children with severe acute diarrhoea within the specified age range who appeared to be high-risk at admission were considered for the trial. Severe acute diarrhoea was defined as acute watery diarrhoea associated with some or severe dehydration, or severe stunting, or moderate wasting [23]. All the dehydrating children were managed at hospital as per treatment protocol before enrolment [24], after which they were followed up for 90 days.

We based our secondary analysis on a case-control design using data from the ABCD trial’s site in Bangladesh. We chose children who required post-discharge re-hospitalisation within a 90-day follow-up period as the cases and randomly selected age- and sex-matched children as the controls from those who did not require readmission after discharge from the hospital during a 90-day follow-up period at a 1:3 ratio. We conducted the random selection of children using the ‘FUZZY’ case-control matching method in SPSS, version 26 (IBM, SPSS Inc., New York, USA) to address the challenges of finding suitable controls for our cases based on variables such as gender and age. We specified a tolerance level of zero for sex and 0.2 for age, indicating that we permitted no deviations for sex, but minor deviations for the age to maximise the number of appropriate controls. We aimed for a 1:3 ratio in case-control matching to ensure an adequate sample size for statistical analyses.

### Variables of interest

We obtained the outcome variables from nutritional indicators: height for age/length for age (HAZ/LAZ), weight-for-age (WAZ), weight-for-height/weight-for-length (WHZ/WLZ) and mid-upper arm circumference (MUAC). Specifically, the variables of interest were the changes in anthropometric indices (ΔHAZ, ΔWAZ, ΔWHZ, and ΔMUAC), whereby the delta denotes the difference between the day-90 follow-up and the enrolment measures. Post-discharge readmission was the main exposure variable for our interest in this analysis.

Based on a thorough literature review, previous studies, and the availability of data in the ABCD trial, we considered several factors as explanatory variables: clinical characteristics on admission, breastfeeding and immunisation status, parent’s characteristics (*e.g*. mother’s age and body mass index (BMI), parent’s education level), household characteristics (*e.g.* type of toilet, water sources, floor type, presence of domestic animals), and the presence of *Escherichia coli* in the faeces.

### Data collection

We obtained anthropometric measurements, including weight, length/height, and MUAC, for both the initial enrolment and 90-day follow-up assessment. We also collected demographic and clinical information, including age, sex, characteristics of the diarrhoea episode, breast-feeding, dehydration at admission, length of hospital stay, clinical outcomes, mortality status, vaccination history, parental education, maternal age, maternal body weight and height, and household characteristics such as building material, water source, sanitary facility, collection of stool samples to identify the presence of *Escherichia coli* [25].

The study protocol details the data collection process [23]. In summary, data were gathered during the initial enrolment phase and subsequent follow-up assessments. The determination of vital status was made based on caregiver reports and hospital records. Standardised protocols were used to collect anthropometric measurements at enrolment and on day 90. Two independent observers measured each anthropometric measurement. Weight was measured with an Electronic Baby Weighing Scale (M118600, ADE, Germany) with a sensitivity of 10g, while length was measured with a Baby Length Measuring Board (MZ10040, ADE, Germany) to the nearest 0.1 cm. MUAC was measured with a non-stretchable tape. For children over two years of age (during follow-ups), height was measured to the nearest 0.1 cm using a roll-up measuring tape with a wall attachment. All instruments were calibrated daily, with weight and length measured by two independent observers and averages of three readings recorded. Measurements were taken post-rehydration and stabilisation, following World Health Organization (WHO) standards. Data collectors received rigorous training and regular refresher courses, and data quality was maintained through blinded collection, controlled environments, and electronic data capture systems (REDCap) with consistency checks.

### Operational definition

#### Ponderal and linear growth

Ponderal growth refers to the change in weight relative to height, often expressed through measures like the ponderal index or WHZ/WLZ. It assesses the balance between a child's weight gain and their height increase, providing insight into their overall growth and nutritional status. Linear growth refers to the increase in height or length over time, and is a key indicator of overall child health and nutritional status.

#### Post-discharge re-hospitalisation

The ABCD trial defined post-discharge re-hospitalisation if the enrolled children were readmitted to any hospital after being discharged from the study facility, specifically within 90 days [25]. These re-hospitalisations were monitored through caregiver reports at each study visit.

#### Ethical consideration

The ABCD trial received approval from both the World Health Organization (WHO) Ethics Review Committee and the local Institutional Review Board, the icddr,b Ethics Committee. Before enrolment, parents or caregivers were provided with comprehensive information about the ABCD trial, including its purpose, procedures, potential risks and benefits. They were allowed to ask questions and discuss any concerns they might have. Written informed consent was obtained properly from the parents or caregivers of all participants, ensuring that their agreement was fully voluntary and based on a clear understanding of the study.

#### Statistical analysis

The ABCD trial collected data on paper forms and subsequently entered it into REDCap, a widely used electronic data capture system, at convenient internet access points. They used the software’s in-built quality assurance module to set up quality control measures, thus ensuring data accuracy and completeness. Once data cleaning was finalised, the data set was exported to Stata, version 15 (StataCorp LLC, College Station, Texas, USA) for statistical analysis.

We used STATA and SPSS, version 26 (IBM, SPSS Inc., New York, USA) for data management and analysis. We summarised descriptive statistics using frequencies and percentages for categorical variables and means with standard deviations or medians with interquartile ranges for continuous variables. We determined the statistical association using the χ^2^ test or Fisher exact test for the categorical variables and the *t* test or Mann-Whitney U test as appropriate for the continuous variables. We first performed a bivariate analysis to explore the associations between baseline characteristics and study groups; afterwards, we used factors that were significantly associated with the grouping variable in our regression models.

The parent study, the ABCD trial, adopted a rigorous methodology that resulted in minimal data gaps. In this secondary analysis, most response variables had completed data (<1% missing) and no imputation was necessary. Anthropometric measurements for six participants were not obtained due to mortality or loss to follow-up; however, all other outcome variables, including age, sex, weight, height, and MUAC, as well as exposure variables, were complete without any missing values. Given the negligible rate of missing data, we adopted a straightforward complete-case analysis approach, excluding observations with missing values from the analysis.

To explore whether any dissimilarity existed in anthropometric changes over time between children with and without post-discharge re-hospitalisation groups, we used the following anthropometric indicators: ΔHAZ, ΔWAZ, ΔWHZ, and ΔMUAC. Each of these outcome variables was subjected to regression analysis against the exposure variable. To assess the relationships between post-discharge hospitalisation and anthropometric changes after the proposed follow-up, we examined them with multiple linear regression models, while accounting for potential covariates. We adjusted all models for covariates such as age, sex, admission z-scores, MUAC, parents’ education, mother’s BMI, water source, admission dehydration, intervention, vaccination, and breastfeeding status. We considered a *P*-value <0.05 as statistically significant.

We selected variables through an extensive examination of existing literature, previous studies, and exploration study of our data prior to constructing the multivariate linear regression models. We used visual techniques (plots and correlation matrices) and unadjusted models to find relevant predictors. After selecting the variables, we assessed the model’s fit using metrics such as the adjusted coefficient of determination (R^2^), the Akaike information criterion (AIC), and the Bayesian information criterion (BIC). To evaluate the quality of the model, we performed residual analysis, verifying that the residuals exhibited a normal distribution and were randomly dispersed. We also assessed multicollinearity by computing the variance inflation factor (VIF). A VIF score of more than five was regarded as a sign of multicollinearity, which might be addressed by eliminating or merging variables, or by employing dimensionality reduction methods such as principal component analysis (PCA). However, these steps were unnecessary in our final models, as all VIF values were <3.

## RESULTS

### Baseline characteristics

**Figure 1:**
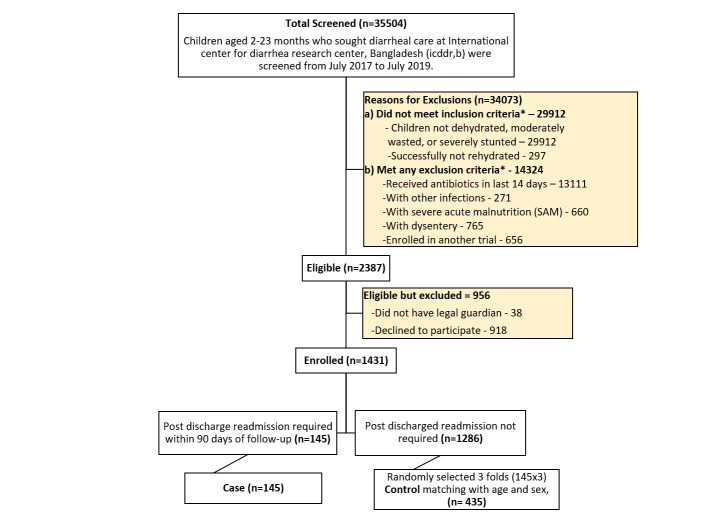
Flowchart of study participants. *Multiple inclusion and exclusion criteria were possible.

The ABCD trial enrolled 1431 children with severe diarrhoea in the Bangladesh site; 145 fit our criteria for cases, and we selected 435 as controls, in line with our desired 1:3 ratio (**Figure 1**). In terms of baseline admission characteristics, the cases were less likely to be immunised and more often had vomiting and subsequent dehydrating diarrhoea than the controls (**Table 1**). Other baseline admission characteristics were comparable between the groups.

**Table 1:** Baseline admission characteristics of cases and controls*

	Case	Control	*P*-value
**Number of participants**	145	435	
**Admission information**			
Sex (male)	92 (63.4)	276 (63.4)	1.000
Age in months, x̄ (SD)	9.99 (5.07)	10.01 (5.03)	0.960
Length/height, x̄ (SD)	68.19 (6.5)	67.93 (6.31)	0.673
Weight, x̄ (SD)	6.89 (1.33)	6.8 (1.25)	0.434
MUAC, x̄ (SD)	12.69 (0.88)	12.64 (0.82)	0.581
WAZ, x̄ (SD)	−2.09 (0.93)	−2.20 (0.85)	0.159
HAZ, x̄ (SD)	−1.56 (1.23)	−1.69 (1.13)	0.249
WHZ, x̄ (SD)	−1.59 (0.96)	−1.64 (0.94)	0.523
Breastfeeding status (exclusive and mixed)	128 (88.3)	401 (92.2)	0.150
Immunisation as per EPI schedule	94 (72.3)	320 (81.2)	0.031
**Clinical characteristics on admission**			
Some/severe dehydration	52 (35.9)	114 (26.2)	0.026
Cough	10 (6.9)	25 (5.7)	0.615
Child vomits everything	32 (22.1)	47 (10.8)	0.001
Fever	10 (6.9)	25 (5.7)	0.615
Abnormal respiratory rate	2 (1.4)	1 (0.2)	0.095
Fast breathing	2 (1.4)	2 (0.5)	0.247
**Mother's age in years**			0.441
>18	3 (2.1)	10 (2.3)	
18–29	105 (72.4)	336 (77.2)	
30≤	37 (25.5)	89 (20.5)	
**≥2 under-five children in the household**	27 (18.6)	69 (15.9)	0.534
**Mother’s BMI, x̄ (SD)**	22.8 (4.5)	22.5 (4.5)	0.411
**Mother’s schooling years**			0.021
No education	21 (14.5)	79 (18.2)	
Below primary	14 (9.7)	77 (17.8)	
Primary and above	110 (75.9)	277 (64.0)	
**Father’s schooling years**			0.436
No education	24 (16.6)	92 (21.3)	
Below primary	18 (12.4)	46 (10.7)	
Primary and above	103 (71.0)	294 (68.1)	
**Household information**			
Toilet with sewer system	117 (80.7)	335 (77.2)	0.378
Piped water source	126 (86.9)	392 (90.1)	0.277
Cemented floor	143 (98.6)	415 (95.4)	0.079
Presence of any animal (cow, goat, duck, quells, chicken, pigeon)	9 (6.2)	33 (7.6)	0.579
*Escherichia coli* found in stool*	23 (100)	97 (95.1)	0.278

BMI – body mass index, EPI – Expanded Programme on Immunization, HAZ – height for age Z-score, MUAC – mid-upper arm circumference, SD – standard deviation, WAZ – weight for age Z-score, WHZ – weight for height Z-score, x̄ – mean

*Values presented as n (%) unless specified otherwise.

### Post-discharge readmitted cases

**Figure 2:**
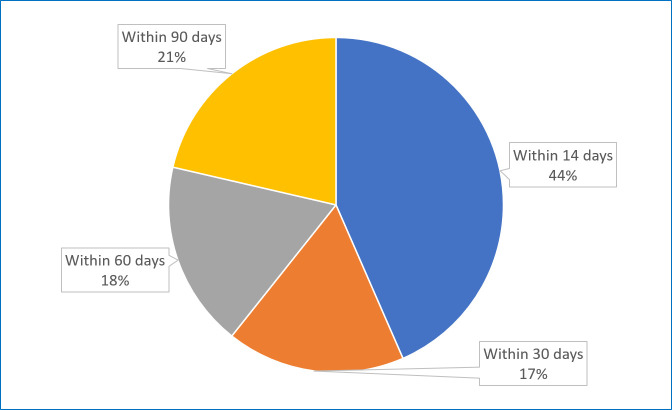
Time points of post-discharge readmission following enrolment.

Among the cases, younger age (<1 year) (71%), and male gender (63.5%) were linked with greater readmission rates (Table S1 in the **Online Supplementary Document**). The first leading cause of readmission was a second or ongoing diarrhoea episode, while the second was respiratory problems. Nearly half of the cases (44%) were readmitted within 14 days of their initial discharge during follow-up (**Figure 2**). After the first hospital discharge, 85% of infants among cases had one more hospitalisation, while others visited two or three times (Table S1 in the **Online Supplementary Document**). Five children (3.5%) died during the follow-up period; one death occurred within two weeks of discharge, another within a month, two more in the second month, and one in the third month. These deaths included those with persistent diarrhoea, sepsis, severe pneumonia with sepsis, probable disseminated intravascular coagulation, and nephrotic syndrome (**Table 3**).

**Table 3:** Deaths among the post-discharged readmission cases

	n (%)
**Total deaths**	5 (3.4)
**Sex**	
Male	3 (2.1)
Female	2 (1.4)
**Age in months, x̄ (SD)**	6.3 (2.2)
**Death time point**	
Within 2 weeks	1 (1.4)
Within 30 d	1 (1.4)
Within 30–60 d	2 (2.1)
Within 61–90 d	1 (1.4)
**Average death time from enrolment (days), x̄ (SD)**	38 (25)
**Diagnosis**	
Persistent diarrhoea	1 (1.4)
Severe pneumonia with sepsis	1 (1.4)
Sepsis	1 (1.4)
Suspected DIC	1 (1.4)
Nephrotic syndrome, persistent diarrhoea, DIC	1 (1.4)

DIC – disseminated intravascular coagulation, SD – standard deviation, x̄ – mean

### Changes in nutritional status at follow-up

**Figure 3:**
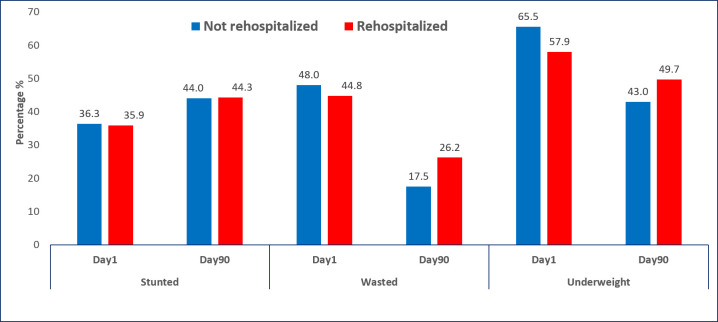
Changes in nutritional indicators over time for children requiring re-hospitalisation compared to those not requiring re-hospitalisation.

The nutritional status of post-discharge readmitted and non-readmitted children was similar during enrollment (**Table 1**). After 90 days, both groups had more stunted children than admission (**Figure 3**), with no statistical difference. Although both wasting and underweight decreased in both groups, the readmitted group had a higher rate of wasting and underweight children. Wasting was significantly different in cases (26.2% *vs.* 17.5%; *P* = 0.016), while the difference in underweight prevalence was not statistically significant.

### Association of the changes in anthropometric indices and re-hospitalisation

To determine the relation between changes in growth and post-discharge re-hospitalisation in children between groups, we analysed four models using anthropometric indices (ΔHAZ, ΔWAZ, ΔWHZ, and ΔMUAC) (**Table 2**). All nutritional indicator models with covariate adjustments showed substantial growth differences. Readmitted children showed a significant decrease in ΔHAZ of −0.11 units (95% confidence interval (CI) = −0.21, −0.01; *P* = 0.029), indicating a shorter height relative to their age. The ΔWAZ indicated a significant drop of −0.24 units (95% CI = −0.35, −0.14; *P* < 0.001), while ΔWHZ also showed a significant decrease of −0.25 units (95% CI = −0.39, −0.12; *P* = <0.001). Additionally, compared to the non-readmitted children model for ΔMUAC among the six months and older, readmitted children showed a significant reduction of −0.17 units (95% CI = −0.29, −0.04; *P* = 0.011).

**Table 2:** Multivariate linear regression models to investigate the difference in anthropometric changes over a 90-d follow-up between case and control*

	ΔHAZ		ΔWAZ		ΔWHZ		ΔMUAC†	
	**Unadjusted β (95% CI)**	**Adjusted β (95% CI)**	**Unadjusted β (95% CI)**	**Adjusted β (95% CI)**	**Unadjusted β (95% CI)**	**Adjusted β (95% CI)**	**Unadjusted β (95% CI)**	**Adjusted β (95% CI)**
**Not readmitted**	ref	ref	ref	ref	ref	ref	ref	ref
**Post-discharge readmitted**	−0.11 (−0.21, −0.01)	−0.11 (−0.21, −0.01)	−0.22 (−0.33, −0.12)	−0.24 (−0.35, −0.14)	−0.21 (−0.36, −0.07)	−0.25 (−0.39, −0.12)	−0.16 (−0.30, −0.02)	−0.17 (−0.29, −0.04)
***P*-value**	0.034	0.029	<0.001	<0.001	0.004	<0.001	0.014	0.011

BMI – body mass index, CI – confidence interval, HAZ – height for age Z-score, MUAC – mid-upper arm circumference, ref – reference, WAZ – weight for age Z-score, WHZ – weight for height Z-score

*All four models were adjusted for baseline anthropometry (HAZ, WAZ, WHZ, MUAC), age, sex, parents’ education, mother’s BMI, water source, admission dehydration, intervention, vaccination, and breastfeeding status.

†For the ΔMUAC model children aged <6 months were excluded.

## DISCUSSION

We found re-hospitalisation had a significant influence on growth impairment, nutritional disparity, and increased rates of morbidity and mortality among children in a 90-day follow-up period. The differences in various growth indicators, like ΔHAZ, ΔWAZ, ΔWHZ, and ΔMUAC significantly declined in re-hospitalised cases, suggesting re-hospitalisation may act as a marker of underlying vulnerabilities with growth impairment, leading to an increased risk of morbidity and mortality. The findings align with prior studies that have demonstrated an association between impairment in growth and serious outcomes in young children [26–28].

The deviations in various growth indicators leading to malnutrition can make a child more vulnerable to infection, while infection further contributes to malnutrition, creating a vicious cycle [29]. The distribution of nutritional status between the re-hospitalised and non-re-hospitalised groups was similar during the enrollment phase (on day one) in our study. However, during the 90-day follow-up, both groups experienced an increase in stunting status, but the difference was not statistically significant. Interestingly, the re-hospitalised group had higher rates of wasting and underweight compared to the non-re-hospitalised group. The difference in the prevalence of wasting was statistically significant, indicating a greater risk of malnutrition in the re-hospitalised group. Being nutritionally wasted in children increases the risk of the worst outcome mortality and other negative consequences [30]. In 2011, over 800 thousand under-five deaths were attributed to wasting [4], and it had previously been found to be a stronger determinant of mortality than stunting or underweight [31]. In our study, we found the difference in underweight prevalence was higher in the readmitted group, albeit this finding was not statistically significant.

Although we observed a low number of deaths among cases (n = 5, 3.4%), two occurred within the initial 30 days of post-discharge, two were reported in the second month, and the remaining one in the third month. These outcomes are consistent with prior studies [32–37], one of which observed the highest mortality rate in the initial month following hospital discharge [35], while another looking at aggregated data for the first three months of post-discharge deaths also found them to be a critical period [34]. These findings bring attention to the vulnerability of patients in the early window following hospital discharge. The higher risk of possible adverse outcomes during the initial days of the post-discharge period demands a strategic focus on interventions and close monitoring during this critical timeframe.

It is imperative to investigate the potential reasons for re-hospitalisation of our study populations for designing future interventions that may help to reduce re-hospitalisation and subsequent growth retardation and deaths during post-discharge follow-up. Over 60% of re-hospitalisation cases were readmitted either due to a continuation of their initial episode of diarrhoea or the onset of a second episode. Another major reason for readmission in our sample were acute respiratory infections, which is also consistent with the scenarios of other LMICs [38–40]. However, a large number of deaths were attributed to pneumonia, which had caused 14% of under-five mortality in 2019 [41]. A substantial proportion (37%) of readmissions occurred within the first 14 days of their follow-up period after initial discharge, underscoring the critical nature of the immediate post-discharge period and highlights the imperative for close monitoring during this time frame. Early readmission can signal potential complications or unresolved issues that require attention, making the first two weeks a crucial window for intervention.

The observation of a lower immunisation rate in post-discharge re-hospitalised children compared to their counterparts emphasises the importance of continuing vaccination per the Expanded Programme on Immunization (EPI) schedule to reduce the likelihood of severe illness leading to re-hospitalisation. Lack of immunisation was previously found to be an important risk factor for death [33]. The global impact of vaccination in preventing disease, disability, and mortality further draws attention to the importance of adhering to the recommended EPI schedule [42,43]. Proper vaccination may play a vital role in mitigating post-discharge hospitalisation risk, contributing to improved public health outcomes, such as saving children from growth retardation, as well as millions of lives every year due to the established protection against an array of infectious diseases [44,45].

We observed that diarrhoeal children with dehydration on admission had a higher prevalence of re-hospitalisation compared to their counterparts. Severe dehydration was found to be associated with mortality in another study [46]. These findings highlight the need to address the severity of dehydration at the time of admission to treat the condition with the appropriate care, which may be essential in preventing subsequent hospital readmissions.

Several limitations of our study need to be acknowledged. The shorter follow-up time may limit long-term trends and results related to mortality and morbidity. We did not address all the socioeconomic factors that may affect post-discharge outcomes. Specifically, we did not include economic indicators such as income, food security, or food diversity in this secondary analysis, as they were unavailable in the ABCD trial. This might have introduced confounding bias in our results. We must also acknowledge that our analysis is limited to data collected from only one out of the multiple countries involved in the original trial. This may have introduced regional bias, as anthropometric changes and responses to antibiotics could vary across different populations. Additionally, the focus on a single country may not have fully captured the variability present in the global trial data, affecting the global generalisability of our findings. However, this analysis is strengthened by the ABCD trial's rigorous and randomised data, obtained from the world's largest diarrheal disease hospitals. This allowed us to evaluate re-hospitalisation characteristics in detail and with reliability during a 90-day post-discharge follow-up. By including anthropometric data and vital status, the ABCD trial experiment provided a holistic perspective of participants' health. Our results are further made more reliable by our following of the WHO norms for evaluating consistency and comparability. Another important strength of the study is the employment of fuzzy matching (SPSS-specific case-control method), which enhanced statistical power, reduced bias, and maintained study integrity, ensuring robust and applicable findings for the population under study.

## CONCLUSIONS

We found that children hospitalised during post-discharge follow-up experienced significantly reduced growth across all anthropometric indices compared to those who were not hospitalised. Our findings highlight the complex impact of post-discharge hospitalisation on growth outcomes in children under two years of age. To mitigate such poor outcomes, it is crucial to implement interventions addressing vaccination, dehydration, and nutritional status during the critical post-discharge period. Further research is needed to investigate long-term trends and the role of socioeconomic factors in post-discharge hospitalisation, which could enhance our understanding and inform more effective interventions in this vulnerable population.

## Additional material


Online Supplementary Document
