# Supplementary material for: Consequences of post-discharge hospitalisation on the growth of young Bangladeshi children hospitalised with diarrhoea: a secondary case-control analysis of Antibiotics for Children with Diarrhea (ABCD) trial
Source: J Glob Health. 2025 Feb 14;15:04039. doi: 10.7189/jogh.15.04039 (PMC11893141; doi:10.7189/jogh.15.04039)
Supplement: Online Supplementary Document [file jogh-15-04039-s001.pdf]

Supplement to: Kabir MF, Parvin I, Shahid ASMSB, Das R, Ackhter MM, Alam T, Khanam S, Sultana J, Nasrin S, Sharmin R, Ahmed MT, Kamal M, Faruk MT, Nuzhat S, Afroze F, Ahmed T, Chisti MJ. Consequences of post-discharge hospitalisation on the growth of young Bangladeshi children hospitalised with diarrhoea: a secondary case-control analysis of Antibiotics for Children with Diarrhea (ABCD) trial. J Glob Health. 2025;15:04039.

Table S1: Post discharged readmission cases and their reasons (n=145)

	Freq.	Percent (%)
**Sex**		
Male	92	63.5
Female	53	36.6
**Age of the children**		
< 1 year	103	71.0
1 year and above	42	29.0
Admission Age (Months) [Mean±SD]	10.0±5.1	
**Number of times readmitted**		
Once	123	84.8
Twice	19	13.1
Three or More	13	2.1
**Reasons:**		
Abdominal Distention	1	0.69
Bronchiolitis with Iron deficiency anemia	1	0.69
Diarrhea	93	64.14
Fever / Enteric Fever / High grade fever	9	6.21
Hematuria	1	0.69
Measles	3	2.07
Nephrotic Syndrome	1	0.69
Early onset of Hospital Acquired Pneumonia	1	0.69
Pneumonia or ARI Respiratory tract infection	25	17.24
Pneumonia with AWD	2	1.38
Pneumonia with Measles	2	1.38
Pneumonia with SAM	1	0.69
Seizure disorder with Hospital Acquired pneumonia	1	0.69
Sepsis with Invasive diarrhea with measles	1	0.69
Severe Acute Malnutrition with bipedal oedema	1	0.69
Severe Pneumonia with Sepsis	1	0.69
Urinary Tract Infection (UTI)	1	0.69
